# Altered Glutaminase 1 Activity During Neurulation and Its Potential Implications in Neural Tube Defects

**DOI:** 10.3389/fphar.2020.00900

**Published:** 2020-06-19

**Authors:** Camila Benavides-Rivas, Lina Mariana Tovar, Nicolás Zúñiga, Ingrid Pinto-Borguero, Claudio Retamal, Gonzalo E. Yévenes, Gustavo Moraga-Cid, Jorge Fuentealba, Leonardo Guzmán, Claudio Coddou, Luisa Bascuñán-Godoy, Patricio A. Castro

**Affiliations:** ^1^Laboratory of Physiology and Pharmacology for Neural Development, LAND, Departamento de Fisiología, Facultad de Ciencias Biológicas, Universidad de Concepción, Concepción, Chile; ^2^Departamento de Fisiología, Facultad de Ciencias Biológicas, Universidad de Concepción, Concepción, Chile; ^3^Departamento de Ciencias Biomédicas, Facultad de Medicina, Universidad Católica del Norte, Coquimbo, Chile; ^4^Departamento de Botánica, Facultad de Ciencias Naturales y Oceanográficas, Universidad de Concepción, Concepción, Chile

**Keywords:** neurulation, neural tube defects, glutamate, glutaminase 1, DON

## Abstract

The neurulation process is regulated by a large amount of genetic and environmental factors that determine the establishment, folding, and fusion of the neural plate to form the neural tube, which develops into the main structure of the central nervous system. A recently described factor involved in this process is glutamate. Through NMDA ionotropic receptor, glutamate modifies intracellular Ca^2+^ dynamics allowing the oriented cell migration and proliferation, essentials processes in neurulation. Glutamate synthesis depends on the mitochondrial enzyme known as glutaminase 1 (GLS1) that is widely expressed in brain and kidney. The participation of GLS 1 in prenatal neurogenic processes and in the adult brain has been experimentally established, however, its participation in early stages of embryonic development has not been described. The present investigation describes for the first time the presence and functionality of GLS1 in *Xenopus laevis* embryos during neurulation. Although protein expression levels remains constant, the catalytic activity of GLS1 increases significantly (~66%) between early (stage 12) and middle to late (stages 14–19) neurulation process. Additionally, the use of 6-diazo-5-oxo-L-norleucine (L-DON, competitive inhibitor of glutamine-depend enzymes), reduced significantly the GLS1 specific activity during neurulation (~36%) and induce the occurrence of neural tube defects involving its possible participation in the neural tube closure in *Xenopus laevis* embryos.

## Introduction

The neurulation process corresponds to a series of complex morphogenetic events which begin around the third week and extends until the fourth week of pregnancy in humans (Blom, 2009). During neurulation, the neural plate cells differentiate, fold, and fuse to form a tubular structure. This structure, called the neural tube, will eventually develop into the brain and spinal cord (Davidson and Keller, 1999; Copp and Greene, 2010; Nikolopoulou et al., 2017). Neurulation disturbances prevent normal neural tube closure and cause neural tube defects (NTDs) (Stottmann et al., 2006; Wallingford et al., 2013). NTDs are one of the most common birth malformations in the human population with a prevalence of 1 per 1,000 births worldwide (Stottmann et al., 2006; Ybot-Gonzalez et al., 2007; Copp and Greene, 2010). Despite the clinical relevance of these diseases, their molecular and cellular causes remain poorly understood (Wallingford et al., 2013). Therefore, an understanding of the neurulation mechanism is essential to explain the embryonic pathogenesis of NTDs.

It is well described that Wnt ligand and Bone Morphogenic protein (BMP) regulates essential processes for neural tube closure, such as planar cell polarity (PCP), convergent extension and formation, apposition, and fusion of neural folds (Reichert et al., 2013; Nikolopoulou et al., 2017). A recent study showed that, in addition to these molecules glutamate also plays an important role in neural tube closure (Sequerra et al., 2018). Thus, the presence of glutamate in early stages of development has already been described (Root et al., 2008), as well as the presence of transcripts for proteins involved in neurotransmission such as the vesicular glutamate transporter (VGluT1), vesicle-associated membrane protein (VAMP1), syntaxin 1 (Stx1), and synaptosome-associated protein 25 (SNAP25) (Sequerra et al., 2018). In addition, different types of glutamate receptors have been found during the neural plate stage and early neurulation (Lujan et al., 2005; Root et al., 2008). Particularly, Sequerra et al. demonstrated that knocking down the expression of the GluN1 subunit from the NMDA receptor increases the number of mitotic neural plate cells and perturbs the lateral-medial migration. Taken together, this data suggests the existence of a glutamate-mediated signaling before synaptic communication. This pathway is capable of regulating neural plate cell proliferation and migration during neurulation (Sequerra et al., 2018).

In adult neural tissue, the main glutamate synthesis depends on the mitochondrial enzyme called glutaminase 1 (GLS1) encoded by the GLS1 gene and widely expressed in kidney and brain tissue (Masson et al., 2006; Li et al., 2016). In its inactive state, GLS1 exists as dimers and needs to be oligomerized to form tetramers or higher oligomers with catalytic activity (Li et al., 2016). The active tetramer deamidates glutamine and produces stoichiometric amounts of glutamate and ammonia (Stalnecker et al., 2017). Since it is activated by phosphate, it is commonly known as a phosphate-activated glutaminase (PAG) (Schousboe et al., 2013). The binding of inorganic phosphate is not only important for ensuring that the proper orientation of the catalytic residues occurs, but also ensures optimal product release (Li et al., 2016; Stalnecker et al., 2017).

In addition to its role in excitatory synapses, it has been shown that GLS1 participates in tumoral (Lukey et al., 2016) and endothelial tissue proliferation (Peyton et al., 2018). In the nervous system, GLS1 has also been reported to participate in migration, proliferation, and differentiation of neural stem progenitor cells (NPCs) in neurogenic areas from the adult brain (Wang et al., 2014). However, the role of GLS1 in early stages of neurulation remains unknown. Since GLS1 is the main pathway to synthesize glutamate and this neurotransmitter is expressed in early stages of development, we aimed to investigate the expression and function of GLS1 enzyme during neurulation. Using *Xenopus laevis* embryos, we demonstrate that GLS1 is present during neurulation and is able to transform glutamine to glutamate. We also show that GLS inhibition, using L-DON, a glutamine analog, could be related with alterations in normal neural tube closure and associated to neural tube defects.

## Materials and Methods

### *Xenopus laevis* embryos

According Sive et al. (2007), embryos were generated by *in vitro* fertilization. Adult females from *Xenopus laevis* were injected once (pre-prime) with 50U of human chorionic gonadotropin hormone (hCG) 1–4 days before egg collection. Later, females were injected again (prime) with 300-400U of hCG 24 h before egg collection. Eggs collected were fertilized with a small piece of minced testis. This was considered time 0 of fertilization. Fertilized oocytes were kept in a 10% Marc’s Modified Ringer’s (MMR) saline solution containing (in mM): 10 NaCl, 0.2 KCl, 0.1 MgSO_4_, 0.5 HEPES, 5 EDTA, and 0.2 CaCl_2_. Dejellying of embryos was performed by briefly swirling fertilized eggs in 2% cysteine solution. After dejellying embryos were kept in 10% MMR solution (pH 8) and collected in stages of neurulation: stage 12.5 (early neurulation), stage 14 (middle neurulation) and stage 19 (late neurulation). Developmental stages were recorded according to Nieuwkoop and Faber (1994). Animals were handled according to the Institutional Animal Care and Use Committee guidelines and under an approved animal protocol using humane procedures.

### RT-PCR Conventional and RT-qPCR Assay

#### Total RNA Extraction

Total mRNA extraction was performed using E.Z.N.A^®^HP total RNA kit according to manufacturer’s instructions. *Xenopus laevis* embryos in neurulation stages and adult brain tissue were used in this assay. Samples (30 mg) (15 embryos) were homogenized in 700 µl of GTC lysis buffer. Centrifugation cycles were performed according the Kit’s instructions. Samples were washed with 500 µl of ethanol (70% v/v) and centrifuged at 10,000 rpm during 5 min. Total RNA was suspended in 30 µl of RNase-Free water and quantified by absorbance at 260 nm (NanoQuant infinite 200 PRO, Tekan). RNA purity was measured according to 260/280 relation. Samples with a ratio inferior to 1.9 were discarded. The RNA Integrity was determined by visualization of 18S and 28S ribosomal subunits bands in a denaturing formaldehyde/agarose gel electrophoresis. RNA was stored at −80°C for further use.

#### Total RNA Reverse Transcription (RT)

DNA synthesis was performed using the reverse transcriptase M-MuLV Enzyme Mix from ProtoScript^®^ II First Strand cDNA Synthesis Kit. In a final volume of 20 µl, 1 µg of total RNA was incubated with 0.5 µg of oligo-(dT)_15_, denatured at 70°C during 5 min and placed on ice for 2 min. Later, 400U of M-MuLV Enzyme Mix were added. The samples were incubated during 1 h at 42°C. Last incubation was carried out at 70°C during 5 min. Same transcription protocol was applied to negative controls, but no oligo-dT or M-MuLV Enzyme Mix was added.

#### cDNA Amplification by PCR

cDNA amplification was carried out in a BioRad thermocycler (Icycler) implementing the Biolabs PCR kit (New England BioLabs). A final volume of 25 µl was prepared using 1× of ThermoPol or Standard Taq Reaction Buffer, 0.2 mM from each dNTPs, a set of specific primers, 0.625 U of DNA Taq-polymerase (New England BioLabs) and 1 µl of RT product. The incubation program was 95°C for 5 min, followed by 35 cycles of 95°C for 30 s, 57°C for 30s, 72°C for 30s, and a final extension of 72°C for 5 min. The following specific primers were used: GLS1 forward 5′-GGAGGTGACCAGAGGGTGAA-3′ and reverse 5′-CTACGTGCAAGGCTGTACGA-3′; GeneBank accession no. XM_018239036.1. Primers were designed using Primer-Blast NCBI software (https://www.ncbi.nlm.nih.gov/tools/primer-blast/) to have melting temperature of 58–60°C and generate PCR products of 90–150 bp. The transcription factor Sub-1 was used as endogenous (housekeeping) gene (Mughai et al., 2018) in order to normalize experimental results (Bustin et al., 2009). This assay was performed four times using different batches of embryos samples. Every measure was made in duplicates o triplicates. Sequencing of PCR product was performed using the same primers mentioned before (**Supplementary Figure S3E**).

#### Agarose Gel Electrophoresis

DNA fragments were separated using agarose gels (1%). Agarose gels were prepared with electrophoresis buffer TAE (Tris-acetic acid EDTA; 40 mM Tris-HCl, 30 mM acetic acid, 1 mM EDTA; pH 7.6) and ethidium bromide at 0.5µg/ml (APEX). Molecular weight marker was 100 bp DNA ladder (GeneRuler, Thermo Scientific). To visualize DNA bands in agarose gel a transilluminator equipment was used.

#### RT-qPCR Assay

RT-qPCR reaction was prepared with Brilliant II SYBR Green qPCR Master Mix (Agilent Technologies, Santa Clara, CA, USA). A final volume of 20 µl was prepared with 2 µl of cDNA and 500nM of each primer set. Mx3000P thermocycler (Agilent Technologies, Inc.) was used with the following program: 5 min at 95°C; 40 cycles 95°C for 30s; 55°C during 30s, 72°C for 30s, and a final extension of 72°C during 5 min. After the PCR cycles, the purity of the PCR products was checked by analysis of the corresponding melting curve. Quantification was calculated comparing the number of cycles required to amplify each gene over the threshold cycle (Ct) in relation to the reference gene (Bustin et al., 2009). Mughai et al. (2018) evaluated several genes in developmental stages and they determined that sub-1 is the most appropriate control because its constant level of expression in this specific type of samples. After first quantification, the ΔΔCt was determined by normalization against stage 12.5. Experiments included four biological replicates (embryos from different batches) and three technical replicates for each biological replicate were performed. The following primer was used: Sub-1 forward 5′-AGCAGGAGAAATGAAGCCAGG-3′ reverse 5′-CCGACATCTGCTCCTTCAGT-3′ GeneBank accession no. XM_018235664.1; GLS1 primers used in this assay were the same as for RT-PCR.

### Western Blot Assay

#### Total Protein Extract

*Xenopus laevis* embryos in neurulation stages and adult brain tissue were used in this assay. Total protein extracts were homogenized using protease inhibitor (ROCHE) and sonicated three times on ice at 300 W. Concentration of lysed protein was normalized using Bradford technique and Nanoquat equipment (M200, Infinite).

#### Polyacrylamide Gel Electrophoresis and Electrotransfer

Denaturing gel of acrylamide 12% (SDS-PAGE) were used to separate the protein of interest. Samples were incubated at 95°C during 5 min with loading buffer (62.5 mM Tris-HCl pH 6.8, 2% SDS, 10% glycerol, 0.01% bromophenol blue) and 100 mM DTT. Approximately, 125 µg of total protein were loaded on gel next to the protein standard (Spectra Multicolor, Broad Range Protein Ladder, Thermo Scientific), and run at 100 V in run solution (25 mM Tris, 250 mM glycine, and 0.1% SDS). Protein were transfer to Immobilon-P membrane (0.45 μm pore, Merck Millipore, Tullagreen, Carrigtwohill, Irlanda) with transfer solution (25 mM Tris, 192 mM glycine, 20% methanol) at 250 mM during 2 h.

#### Protein Immunodetection

Several washes with TBS-Tween (150 mM NaCl, 10 mM Tris, 0.05% Tween20) were performed. Membrane was blocked with 5% milk in TBS-Tween during 1 h. Overnight incubation at 4°C using primary antibodies was carried out. Primary antibodies: GLS (1:250, Novusbio), N-cadherin (1:1,000, SySy), and β-actin (1:300, SySy). Secondary antibody incubation was performed during 1 h using anti-mouse and anti-rabbitt peroxidase conjugated antibodies (1:1,000; Jackson ImmunoResearch Laboratories, Inc.). Membrane was washed with TBS-Tween for 10 min three times. Finally, membrane was revealed with chemiluminescent solution (Western Lightening Plus-ECL”, Perkin Elmer) in the chemiluminescent and fluorescent equipment (Licor).

### Mitochondrial Enrichment

To improve the yield of GLS1, a mitochondrial enrichment protocol (Frezza et al., 2007) was applied before enzyme activity measurement. Samples were homogenized using 400 µl of IBc buffer containing: 10 mM Tris-MOPS (0.1 M Tris using MOPS to adjust pH to 7.4), 1 mM EGTA/Tris (0.1 M EGTA using Tris to adjust pH to 7.4), 200 mM sucrose at pH 7.4, and then centrifuged at 600g for 10 min at 4°C. The supernatant was removed and samples were centrifuged again at 7,000g for 10 min at 4°C. The resultant pellet was suspended in 400 µl of IBc buffer and then centrifuged at 7,000g for 10 min at 4°C. Finally, each pellet was homogenized in a 40 µl homogenization buffer (0.1 M EDTA-TRIS (0.1 M EDTA using Tris to adjust pH to 7.4) and 1 unit of protease inhibitor, Roche).

### Enzymatic Activity Measurement

A two-step protocol was used to assay glutaminase activity (Botman et al., 2014; Lukey et al., 2016). In the first reaction, GLS1 transforms glutamine to glutamate, then in a second reaction, the enzyme glutamate dehydrogenase (GDH) catalyses the oxidative deamination of glutamate to form a-ketoglutarate and NADH. In general, one mole of glutamate produces the same amount of NADH, which can be measured through its absorbance at 340 nm at 25°C. For the first reaction assay, 35 µl from homogenized mitochondrial enrichment embryos samples [approximately ~40 µg of total protein (38.8 ± 2.8)] were added to 105 µl of reaction mixture 1 containing: 20 mM glutamine, 0.2 mM EDTA, 50 mM Tris-acetate at pH 8.6. Samples were rocked at 37°C for 45 min. The reaction was then quenched by adding 10 µl of 3 M HCl, and samples were placed on ice. Next, 45 µl of reaction mixture 1 was added to 200 µl of reaction mixture 2 containing 1 unit bovine liver GDH, 80 mM Tris-HCl, 200 mM hydrazine, 0.25 mM of ADP (adenosine diphosphate), 2 mM NAD (Nicotinamide adenine dinucleotide) at pH 9.4. Then, the changes in Absorbance associated with NADH production was measured at 340 nm at 25°C to final point (1 h). We used the endpoint register to calculate the amount of glutamate produced. The values of GLS activity are expressed as nmol of NADH production per min^−1^ per mg^−1^ total protein. Enzymatic activity assay without substrate glutamine (gln) and without the incubation period (45 min at 37°C) was used as blank to evaluate the basal level and glutamate formation independent of GLS *in vitro* activity. These value were substracted to the total activity and the contribution of these blanks to the total activity were always less than 20%

We determined the effect of competitive inhibitor 6-diazo-5-oxo-L-norleucine (also known as L-DON) over GLS1 obtained from *X. laevis*. After mitochondrial enrichment, embryo samples at stage 12.5 were incubated with different concentrations of L-DON (0, 1, and 5mM) during 40 min. A two-step protocol for enzymatic activity was then applied. Enzyme and reagents were acquired from Sigma-Aldrich.

### Protein Quantification

Quantification of total protein obtained from *X. laevis* embryos and kidney samples was carried out using a Micro BCA™ kit following the manufacturer’s instructions. A standard curve was also prepared by adding known amounts of Bovine serum albumin (BSA) (0, 2.5, 5, and 10 µg) to reaction mix. This allowed to determine the protein mass contained in *X. laevis* samples. The absorbance was measured at 560 nm at 37°C.

### Pharmacological Assay

Embryos were incubated with L-DON at different concentrations (1 µM, 3 µM, 10 µM, 30 µM, 100 µM, 300 µM, and 1 mM) and 10% Marc’s Modified Ringer’s (MMR) saline solution was used as control. The incubation started at stage 12.5 (early neurulation) until stage 20. Afterward, embryos were removed from treatment and suspended in 10% MMR solution. The anterior-posterior measure of embryo length was performed at tadpole stage 45-50.

### Data Analysis

Results are expressed as mean ± s.e.m. Statistical analyses were performed using one-way analysis of variance (ANOVA). P < 0.05 was considered statistically significant. All experiments were performed at least six times in duplicate for each trial. Embryos of both sexes were included in this study.

## Results

**Figure 1:**
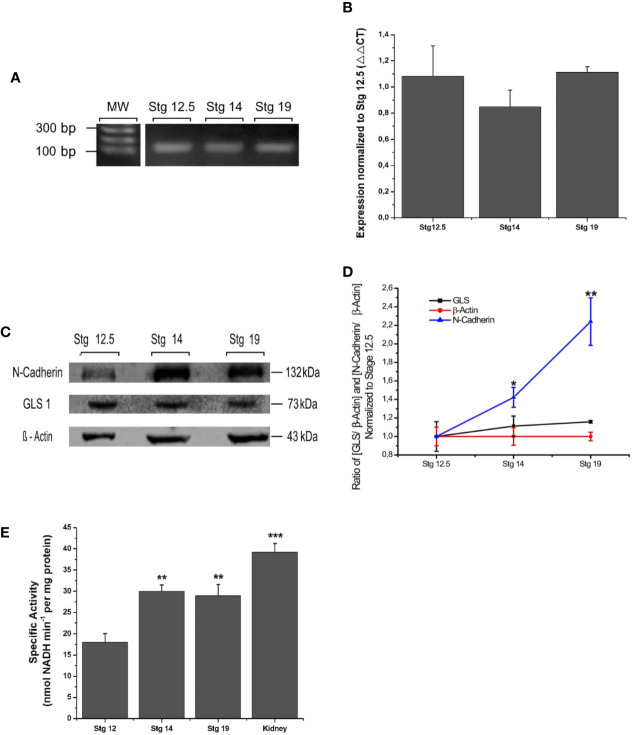
Expression and functionality of GLS1 during neurulation. **(A)** PCR assay revealed the presence of GLS transcripts at all neurulation stages and also in the adult brain (n = 5). **(B)** Transcript quantification using the qPCR assay was normalized to early neurulation. The graph does not show significant differences between stages (n = 6). **(C)** Protein samples obtained from embryos at stg 12.5, stg 14, and stg 19 were analyzed using the western blot assay to determine GLS1 expression (1:250), using β-actin as a loading control (1:300) and N-cadherin (1:1,000) as a neural tissue positive control. The results revealed the presence of protein in all samples (n = 6). **(D)** Quantification of western blot results as ratio [GLS/β-actine] and [N-cadherin/β-actin] demonstrate that GLS1 levels are constant during neurulation. **(E)** GLS1 specific activity expressed as nmol NADH min^−1^ per protein mass (mg). The graph indicates that GLS1 activity increases in stg 14 and remains the same in stg 19, in both cases the activity is significantly lower than kidney, used as a positive control (n = 8). Results expressed as mean ± SEM, *p < 0.05; **p < 0.01; ***p < 0.001. PCR and WB original results were added to **Supplementary Figure S3**.

To examine the expression of GLS1 during neurulation, samples from *Xenopus laevis* were collected at different stages of development: early neurulation (stg 12.5), middle neurulation (stg 14), and late neurulation (stg 19–20). GLS1 expression was measured using conventional PCR, RT-qPCR and western blot assays. Our data showed the presence of transcripts for GLS1 at all neurulation stages (**Figure 1A**). The transcript quantification did not show significant differences between the neurulation stages (**Figure 1B**) (stg 12.5: 1.1 ± 0.2; stg 14: 0.8 ± 0.1; stg 19: 1.1 ± 0.04). To confirm protein expression using the western blot assay, we used β-actin, a cytoskeleton protein, as control, and N-cadherin, an adhesion protein preferentially expressed in neural tissue. The results revealed expression of GLS1 proteins during neurulation (**Figure 1C**), and similar to qPCR data, the protein level did not change significantly during the process (**Figure 1D**) (GLS1: stg 12.5: 1 ± 0.2, stg 14: 1.1 ± 0.1, stg 19: 1.2 ± 0.01; β-actin: stg 12.5: 1 ± 0.1, stg 14: 1 ± 0.1, stg 19: 1 ± 0.04 and N-cadherin: stg12.5: 1 ± 0.006, stg 14: 1.4 ± 0.1, stg 19: 2.2 ± 0.3). Taken together, these results demonstrate that GLS1 is expressed during neural tube formation.

To confirm that GLS1 is a catalytically active enzyme, embryos in neurulation stages were collected and evaluated using an enzymatic assay (see *Materials and Methods*). Again, whole embryos were used. Since GLS1 is widely expressed in kidney we used these samples from adult *X. laevis* as positive control. Our data revealed the presence of glutamate in all the samples analyzed, thereby demonstrating the functionality of GLS1. The activity values of neurulation samples oscillate in the activity range obtained from control groups Glu 0 mM and Glu 0.25 mM (See **Supplementary Figure S1A**).

These results show a significant increase in the activity of GLS1 from the samples at stage 14. This activity remained the same at stage 19, but in both cases was lower than GLS1 activity obtained from kidney samples (**Figure 1E**) (stg 12.5: 18.0 ± 2.1; stg 14: 29.9 ± 1.5; stg 19: 28.9 ± 2.7; and kidney: 39.2 ± 2.03). From these data, we concluded that GLS1 is able to synthesize glutamate from glutamine, and its activity increases during neurulation.

**Figure 2:**
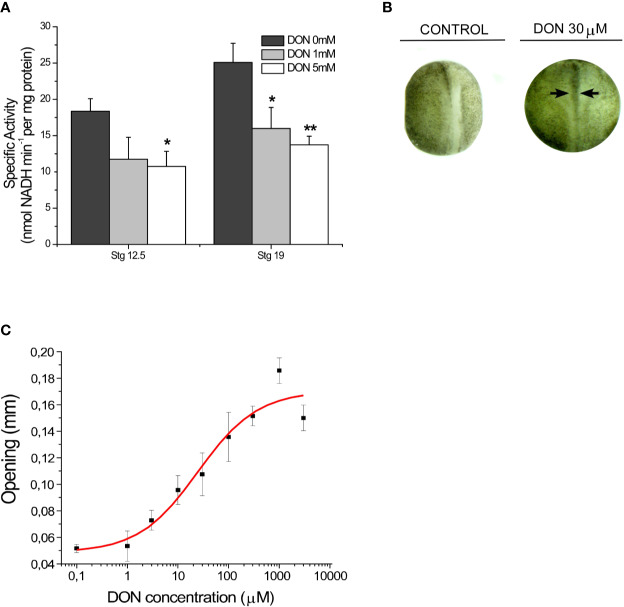
GLS1 inhibition results in a NTDs phenotype in *Xenopus laevis* embryos. **(A)** Inhibition of enzymatic activity of GLS1 using DON in embryo samples at stage 12.5 and stage 19. GLS1 activity is significantly reduced with DON concentrations of 1 and 5 mM (n = 6). DON inhibition in kidney samples was also performed (**Supplementary Figure S1**). **(B)** Sample phenotypes obtained at stg 20 after DON incubation during neurulation, using 0 and 30 µM of DON. Controls were established using a 10% MMR saline solution. After incubation embryos were washed and placed in a 10% MMR saline solution. (n = 6) **(C)** NTDs severity was measured by examining the horizontal opening of the neural tube (black arrows from **B**) after incubation with different concentrations of DON (1 µM, 3 µM, 10 µM, 30 µM, 100 µM, 300 µM, 1 mM, 3 mM, and 10 mM). Controls were established using a 10% MMR saline solution. The maximal severity was obtained with 1mM of DON (0.18 mm opening) (n = 6; EC_50_ of 25 ± 15 µM). (Results expressed as mean ± SEM, *p < 0.05; **p < 0.01.

Finally, the effect of GLS inhibition on embryos phenotype was evaluated. Through *in vitro* assays, we first examined the efficacy of the competitive inhibitor 6-diazo-5-oxo-L-norleucine (L-DON) in *Xenopus laevis* embryos in early and late neurulation. After homogenization process, samples were incubated with 0, 1, and 5 mM of DON during 40 min. The samples were later evaluated with the same enzymatic activity assay. The results showed that DON statistically inhibited GLS activity in embryos on early neurulation (**Figure 2A**) (stg 12.5: DON 0 mM: 18.4 ± 1.7, DON 1 mM: 11.7± 3.02, and DON 5 mM: 10.7 ± 2.1; stg 19: DON 0mM: 25.1 ± 2.6, DON 1 mM: 15.9 ± 2.9 and DON 5 mM: 13.7 ± 1.2). Later, pharmacological assays were carried out incubating embryos at stage 12 with increasing concentrations of DON and 10% MMR solution for controls until stage 20. The severity of NTDs was determined measuring the horizontal opening of the neural tube (**Figure 2B**). The dose-response curve analysis displayed an EC_50_ of 25 ± 15 µM. The maximal effect was obtained with concentrations of 1 mM of DON (0.18 mm opening) (**Figure 2C**). Subsequent analyses showed that DON also altered the anterior-posterior length of embryos at tadpole 45–50 stage (IC_50_ of 124 ± 35 µM) and the curvature of the tail (IC_50_ of 598 ± 221 µM) (**Supplementary Figure S2**).

## Discussion

Neurulation is a critical period in the vertebrate’s development because it determines the establishment of the central nervous system (Christodoulou and Skourides, 2015). This process is the result of highly regulated interactions between growth factors, local gradient concentration, receptor expression, and the presence of morfogenetic neurotransmitters (Luk and Sadikot, 2004). Prenatal exposure to medications that modulate glutamatergic or GABAergic mechanisms, including sedatives, anticonvulsants, and anesthetics, have been associated with aberrations in the proliferation and development of the brain (Luk and Sadikot, 2004). Particularly, the study conducted by Sequerra et al. (2018) showed that glutamate-mediated signaling is essential for neural tube closure and alterations in this pathway result in NTDs in *Xenopus laevis* embryos. Here, we evaluated the presence and functionality of an important component in glutamate-mediated signaling: the GLS1 enzyme. This protein is widely expressed in brain tissue (Masson et al., 2006) and represents the main pathway to synthesize glutamate in neurons (Schousboe et al., 2013). In our experiments, we demonstrated the presence of mRNA and GLS1 protein during neurulation stages in *Xenopus laevis* embryos. However, GLS1 expression remained constant during neural tube closure. Later, the presence of glutamate in *Xenopus laevis* samples revealed the catalytic activity of GLS1. The enzymatic activity assay includes an incubation period which glutamine is transformed into glutamate. Samples without this incubation period produces lower concentration of glutamate. This suggests that the presence of glutamate in neurulation is related with the catalytic activity of GLS and not only come from TCA (tricarboxylic acid cycle) intermediaries. Even when protein expression remains constant during neurulation the catalytic activity of GLS increases significantly (~66%) during the process. Several molecules are able to increase GLS1 activity, one of the most important is inorganic phosphate. This allosteric activator allows the pairing of dimers to form an active tetramer, ensure the proper orientation of the catalytic residues and the optimal product release (Li et al., 2016). In addition to phosphate, ADP is also considered a GLS regulator increasing the affinity with substrate glutamine. This effect is enhanced by the presence of ATP (Massola and Ngubone, 2010).

How GLS1 participates in neurulation is still unclear. However, the most likely alternative is through the immediate product of catalytic activity: glutamate. On one hand, glutamate is transformed into α-ketoglutarate, a TCA intermediary, regulating energy metabolism and promoting cell proliferation, an essential event in neurulation (Lukey et al., 2016; Rumping et al., 2019). Moreover, it was recently discovered that glutamate plays an important role in neurulation, acting through NMDA receptors (Sequerra et al., 2018). NMDA receptors are necessary for normal Ca^+2^ signaling and this ion regulates biomechanical processes and epithelial remodeling during neurulation. Ca^+2^ is also associated with proliferation and migration of neural plate cells (Sequerra et al., 2018) and apical constriction necessary for the elevation of neural folds (Christodoulou and Skourides, 2015; Suzuki et al., 2017). Therefore, GLS1 synthesizes glutamate that participates in neural tube closure.

The GLS loss of function seems to have profound consequences for both construction and maintenance of brain structures (Rumping et al., 2019). Furthermore, a study by Wang et al. (2014) demonstrated for the first time that GLS1 inhibition is detrimental for proliferation, differentiation, and survival of neural precursor cells, but there is no evidence of GLS1 inhibition in early stages of development. Here, we demonstrate that L-DON, a well-documented GLS1 inhibitor, reduce GLS1 specific activity and could participate in the alterations of the neural tube closure. L-DON is a non-selective glutamine analog a capable of interacting with other enzymes such as NAD-synthase, cytidine triphosphate (CTP)-synthetase, and some aminotransferases (Stalnecker et al., 2015; Xu et al., 2019). Consequently, these phenotypic results can be the summary of several inhibitions mediated by L-DON, including GLS1. Fortunately, the establishment of GLS1 as an oncogene (Zhang et al., 2019) has allowed the discovery of more specific inhibitors for the enzyme. It would be interesting to evaluate the effect of these new inhibitors during neurulation in further investigations. Here, we demonstrated that GLS1 is present in early stages of neural development as an active enzyme, but future investigations are needed to elucidate how GLS1 participates in neurulation and how alterations in its activity modify normal neural tube closure.

## Conclusions

Our investigation describes for the first time the presence and functionality of GLS1 at early stages of neural development. Furthermore, pharmacological inhibition with L-DON prevents neural tube closure showing the possible participation of GLS1 in neurulation. Notwithstanding, further investigations using additional strategies will be necessary to understand the fine mechanism of action of this pathway.

## Data Availability Statement

The datasets generated for this study are available on request to the corresponding author.

## Ethics Statement

The animal study was reviewed and approved by Universidad de Concepción.

## Author Contributions

CB-R, LT, NZ, IP-B, CR, and PC performed the research. JF, LG, LB-G, CC, GM-C, GY, and PC designed the research and contributed with analytical tools. CB-R, LT, NZ, IP-B, CR, and PC analyzed the data. CB-R and PC wrote the manuscript. All authors contributed to the article and approved the submitted versión.

## Funding

This work was funded by FONDECYT 11160562 and VRID 220.033.112-4 (PAC). CB-R, LT, and NZ were supported by the Universidad de Concepción postgraduate fellowships (MSc Program in Biochemistry and Bioinformatics).

## Conflict of Interest

The authors declare that the research was conducted in the absence of any commercial or financial relationships that could be construed as a potential conflict of interest.

## Supplementary Material

Supplementary Figure S1 **(A)** Temporal register of enzymatic activity assay. Absorbance was measured at 340nm (as NADH production) for embryo samples (stg 12.5, stg14 and stg 19) and kidney from adult X. laevis (n = 6). Controls were established with different amounts of glutamate (0 and 0.25 mM). The curve shows activity in all embryo samples and higher activity in adult kidney. **(B)** Specific GDH activity of glutamate controls 0 (0.9 ± 0.4) and 0.25 mM (42.9 ± 4.7). **(C)** Inhibition of enzymatic activity of GLS1 using DON in kidney tissue. GLS1 specific activity is significantly reduced with DON concentrations of 1 and 5 mM (n = 6) (0 mM: 37.8 ± 2.1; 1 mM: 23.4 ± 8.2; 5 mM: 21.5 ± 9.7).

Supplementary Figure S2 GLS1 inhibition alters the phenotype of *Xenopus laevis* tadpoles. **(A)** Phenotypes obtained at tadpole stage after DON inhibition during neurulation using 3 µM, 30 µM, 300 µM and 3 mM DON concentration. **(B)** Higher concentrations of DON result in a shortening of tadpole length. The maximal effect was obtained with 1 mM of DON (IC_50_ of 124 ± 35 µM). **(C)** GLS inhibition also alters the tail curvature in a dose-response manner with a maximal effect obtained with 10 mM (IC_50_ of 598 ± 221 µM). (Results expressed as mean ± SEM; n = 6).

Supplementary Figure S3 **(A)** PCR original results. Lane 1 corresponds to molecular weight; lanes 10, 11 and 12 correspond to transcripts from GLS1 obtained from embryo samples at stg 12.5, 14, 19, respectively. All samples were obtained from Xenopus laevis. Lanes 2 to 9 and 13 to 28 correspond to transcripts from other proteins irrelevant to this study. **(B)** Western blot original results for GLS1. Lane 1 corresponds to page ruler; lanes 2, 3 and 4 correspond to GLS1 protein obtained from embryos at stg 12.5, 14 and 19, respectively. **(C)** Western blot original results for β-actin. Lane 1 corresponds to page ruler; Lanes 2, 3 and 4 correspond to β-actin obtained from embryos at stg 12.5, 14 and 19. **(D)** Western blot original results for N-cadherin. Lanes 1 and 5 corresponds to page ruler; Lanes 2, 3 and 4 corresponds to N-cadherin obtained from embryos at stg 12.5, 14 and 19 respectively. **(E)** Chromatogram of Sanger sequencing. A region of PCR product sequence presents a 100% identity with GLS1 mRNA from *Xenopus laevis*. Results were obtained through local alignment of sequences (BLAST Pubmed and Xenbase).
