# Supplementary figures and images for: Altered Glutaminase 1 Activity During Neurulation and Its Potential Implications in Neural Tube Defects
Source: Front Pharmacol. 2020 Jun 19;11:900. doi: 10.3389/fphar.2020.00900 (PMC7316894; doi:10.3389/fphar.2020.00900)

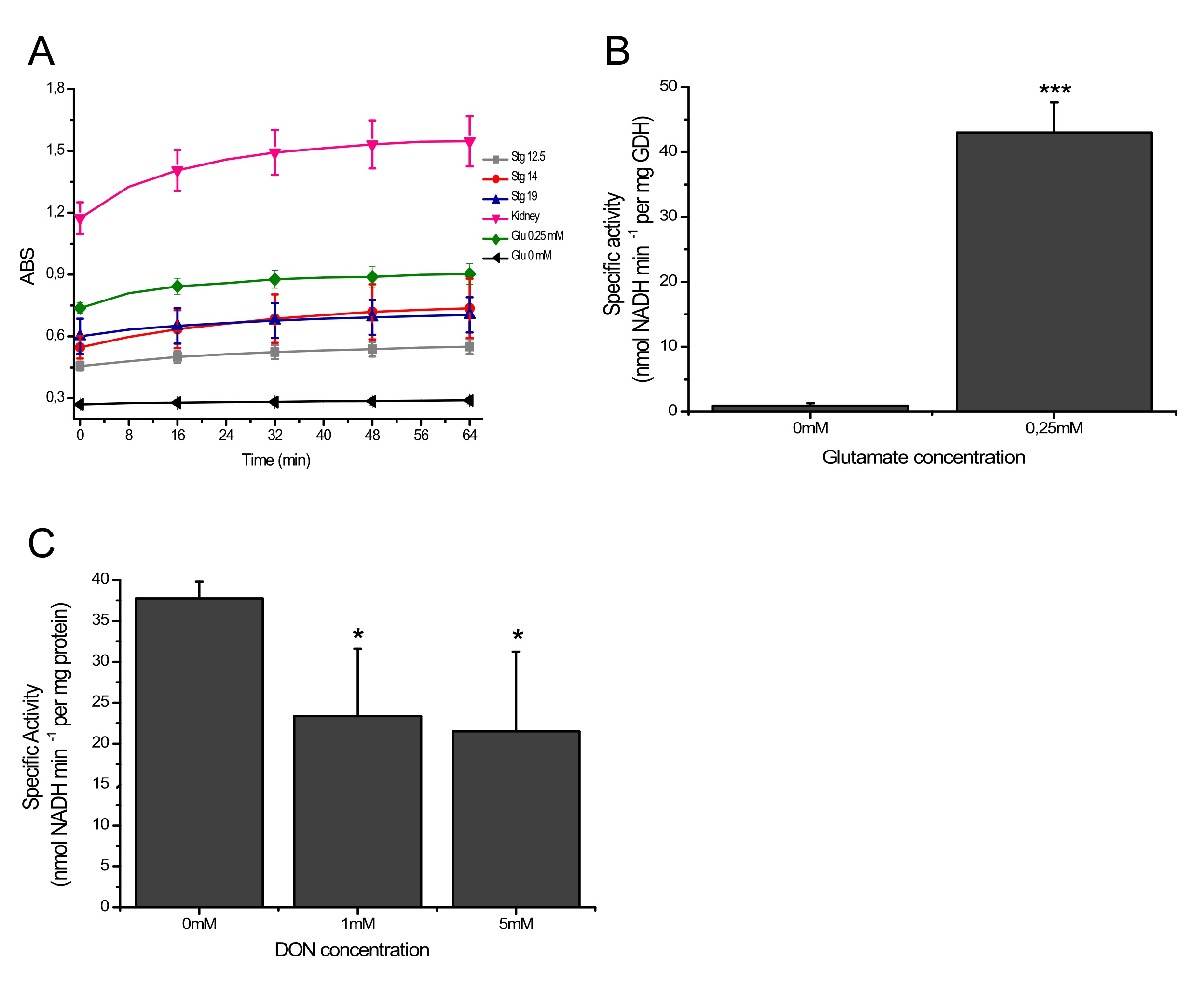

Supplement: Supplementary Figure S1 — (A) Temporal register of enzymatic activity assay. Absorbance was measured at 340nm (as NADH production) for embryo samples (stg 12.5, stg14 and stg 19) and kidney from adult X. laevis (n = 6). Controls were established with different amounts of glutamate (0 and 0.25 mM). The curve shows activity in all embryo samples and higher activity in adult kidney. (B) Specific GDH activity of glutamate controls 0 (0.9 ± 0.4) and 0.25 mM (42.9 ± 4.7). (C) Inhibition of enzymatic activity of GLS1 using DON in kidney tissue. GLS1 specific activity is significantly reduced with DON concentrations of 1 and 5 mM (n = 6) (0 mM: 37.8 ± 2.1; 1 mM: 23.4 ± 8.2; 5 mM: 21.5 ± 9.7). [file DataSheet_1.zip › SUPPLEMENTARY FIGURE 1.jpg]

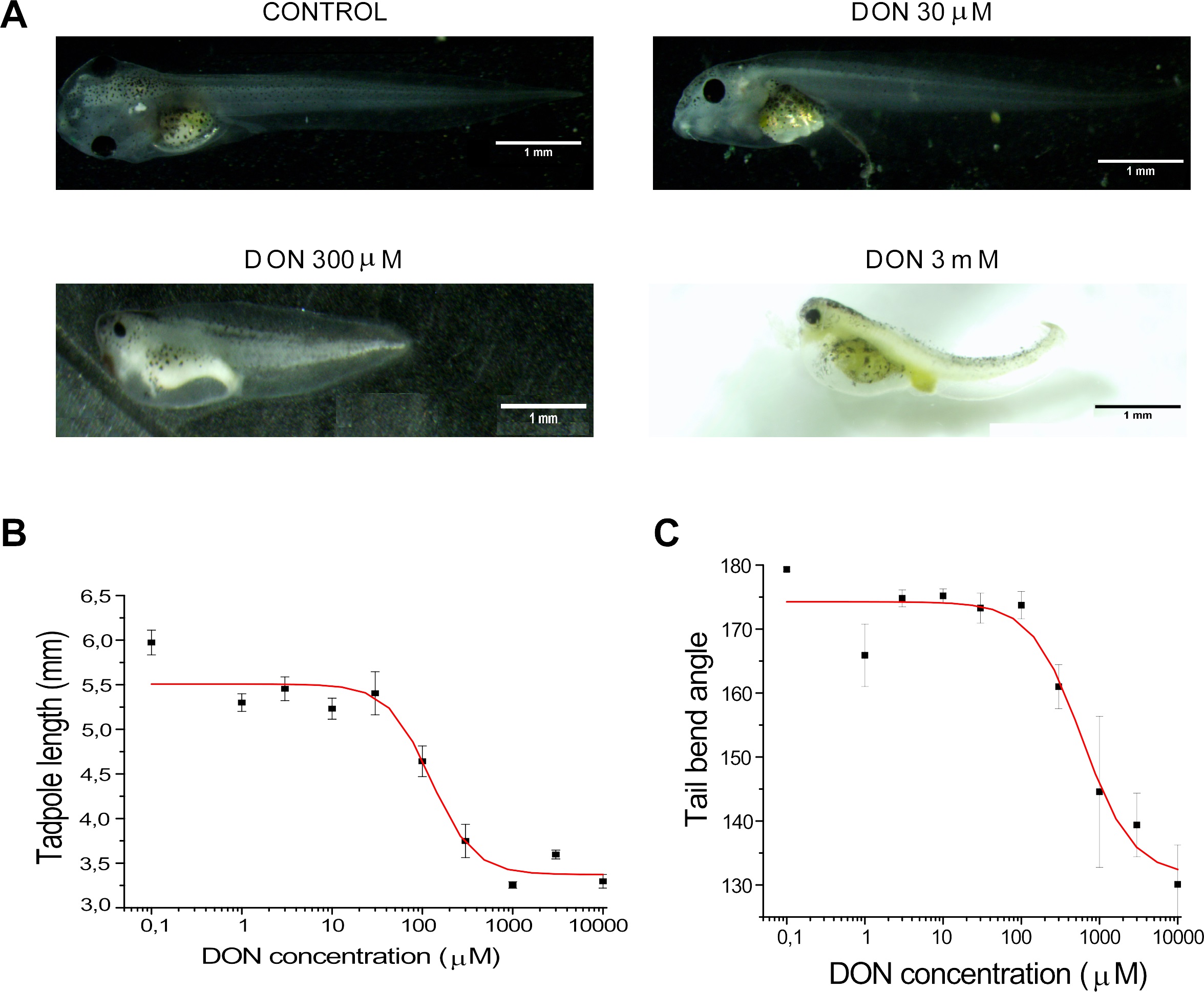

Supplement: Supplementary Figure S1 — (A) Temporal register of enzymatic activity assay. Absorbance was measured at 340nm (as NADH production) for embryo samples (stg 12.5, stg14 and stg 19) and kidney from adult X. laevis (n = 6). Controls were established with different amounts of glutamate (0 and 0.25 mM). The curve shows activity in all embryo samples and higher activity in adult kidney. (B) Specific GDH activity of glutamate controls 0 (0.9 ± 0.4) and 0.25 mM (42.9 ± 4.7). (C) Inhibition of enzymatic activity of GLS1 using DON in kidney tissue. GLS1 specific activity is significantly reduced with DON concentrations of 1 and 5 mM (n = 6) (0 mM: 37.8 ± 2.1; 1 mM: 23.4 ± 8.2; 5 mM: 21.5 ± 9.7). [file DataSheet_1.zip › SUPPLEMENTARY FIGURE 2.jpg]

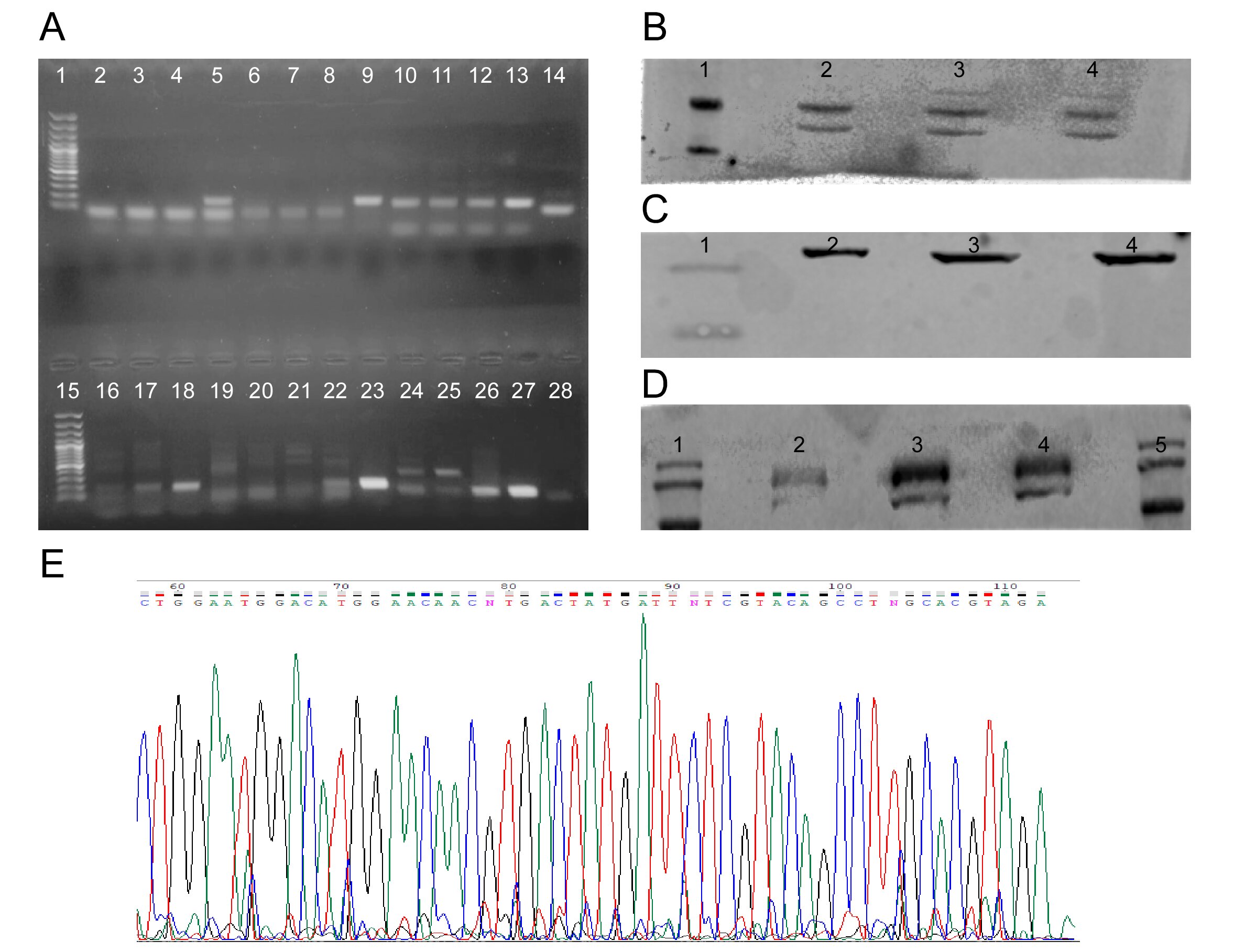

Supplement: Supplementary Figure S1 — (A) Temporal register of enzymatic activity assay. Absorbance was measured at 340nm (as NADH production) for embryo samples (stg 12.5, stg14 and stg 19) and kidney from adult X. laevis (n = 6). Controls were established with different amounts of glutamate (0 and 0.25 mM). The curve shows activity in all embryo samples and higher activity in adult kidney. (B) Specific GDH activity of glutamate controls 0 (0.9 ± 0.4) and 0.25 mM (42.9 ± 4.7). (C) Inhibition of enzymatic activity of GLS1 using DON in kidney tissue. GLS1 specific activity is significantly reduced with DON concentrations of 1 and 5 mM (n = 6) (0 mM: 37.8 ± 2.1; 1 mM: 23.4 ± 8.2; 5 mM: 21.5 ± 9.7). [file DataSheet_1.zip › SUPPLEMENTARY FIGURE 3.jpg]
